# A murine model of adult gastrointestinal colonization by Group B *Streptococcus*

**DOI:** 10.1128/iai.00527-25

**Published:** 2026-01-30

**Authors:** Joie Ling, Luke R. Joyce, Kelly S. Doran, Andrew J. Hryckowian

**Affiliations:** 1 Department of Medicine, Division of Gastroenterology and Hepatology, University of Wisconsin School of Medicine and Public Health 5232, Madison, Wisconsin, USA; 2 Department of Medical Microbiology and Immunology, University of Wisconsin School of Medicine and Public Health 5232, Madison, Wisconsin, USA; 3 Microbiology Doctoral Training Program, University of Wisconsin-Madison 5228, Madison, Wisconsin, USA; 4 Department of Immunology and Microbiology, University of Colorado Anschutz Medical Campus 129263, Aurora, Colorado, USA; University of Illinois Chicago, Chicago, Illinois, USA

**Keywords:** pathobiont, asymptomatic colonization, *Streptococcus agalactiae*, Group B *Streptococcus*

## Abstract

Group B *Streptococcus* (*Streptococcus agalactiae*, GBS) is a leading cause of invasive infections in neonates and adults. The adult gastrointestinal (GI) tract represents an understudied site of asymptomatic carriage with potential relevance for both transmission and disease. Here, we establish a murine model of GBS colonization in the adult GI tract, which provides a tractable system for probing host-microbe interactions within this niche. Using this model, we establish that GI carriage is generalizable to diverse GBS isolates and leverage transposon sequencing (Tn-Seq) to identify candidate GBS factors important for GI colonization. Informed by these Tn-Seq data, we identify GBS capsule as a critical colonization factor of the adult murine GI tract. Taken together, this work highlights the GI tract as a reservoir for GBS and introduces a new experimental framework for investigating the bacterial and host determinants of GBS GI carriage.

## INTRODUCTION

Group B *Streptococcus* (*Streptococcus agalactiae*, GBS) is a disease-causing gram-positive bacterial species. Found asymptomatically in the adult gastrointestinal (GI) tract and the female reproductive tract, GBS causes a wide variety of infections in neonates (1, 2), pregnant people (3), and non-pregnant people (4–6), especially immunocompromised and elderly adults. While preventative interventions decreased the incidence of GBS in neonates, the incidence of GBS disease in non-pregnant adults is increasing worldwide (7). This highlights the limitations of current preventative measures and treatments for GBS and emphasizes the need for the development of new therapeutic strategies against GBS.

The current standard of care for GBS disease prevention and treatment is antibiotics. In the United States, pregnant individuals undergo routine antepartum screening via a rectovaginal swab (8). GBS-positive mothers or mothers with undetermined GBS status are given intrapartum antibiotic prophylaxis to prevent vertical transmission (8). However, there are serious and growing concerns about the sole reliance on antibiotics. First, antibiotics do not completely prevent GBS disease, as intrapartum antibiotics do not impact late-onset GBS disease or recurrence of GBS disease in neonates (9). Similarly, antibiotics also do not completely prevent recurrence of GBS after the end of GBS treatment for non-pregnant adults (10). Second, while penicillin is the first-line antibiotic for intrapartum prophylaxis and for adult infection treatment (8), second-line antibiotics used for those with beta-lactam allergies are not as effective as penicillin in preventing disease or preventing GBS recurrence (11, 12), potentially posing a serious gap in effective care for those with beta-lactam allergies. Concerningly, antibiotic resistance to penicillin and second-line antibiotics is increasing (13, 14), putting all populations at risk.

Finally, there are mounting concerns about the overuse of antibiotics and their known adverse effects, especially in neonates. The use of antibiotics in early life is increasingly connected to short- and long-term adverse effects. Intrapartum antibiotics deplete the maternal vaginal microbiome. Mounting research underscores the importance of this early exposure for proper immune system development in part due to the microbiome (15). Additionally, the use of intrapartum antibiotics is linked to marked changes in the infant microbiome up to a year after birth (16–18). Notable changes include decreases in *Bifidobacterium* spp. (16, 17, 19–21), a keystone species of the developing infant microbiome. As early life exposure to beneficial microbes is linked to the development of the immune system (22), intrapartum antibiotics have potential adverse effects on the infant. In fact, children born to GBS-positive mothers have an increased risk of developing childhood asthma (23).

This study develops a mouse model to begin to understand the understudied GI reservoir of GBS. Notably, GBS is asymptomatically carried in the GI tracts of 15–30% of adults (24). The GI GBS population is a potential source for community spread (9, 25, 26) and can predispose female reproductive tract GBS colonization (27). Targeting GBS in the GI tract first requires a deeper understanding of GBS physiology in this environment. While parallels can be drawn between the GI tract and other body sites where GBS can cause disease, there has yet to be targeted investigation of asymptomatic GBS in the adult mammalian gut. Additionally, this is the first reported *in vivo* model to study GBS in the adult GI tract to our knowledge. Using an *in vivo* transposon sequencing (Tn-Seq) screen, we have begun to identify GBS-encoded factors, such as capsular polysaccharide, that enable long-term GBS GI colonization. Additionally, this model establishes a versatile framework for future study into the bacterial and host factors that shape GBS persistence in the gut.

## RESULTS

### Murine GI carriage of GBS strain COH1

We considered two important host-determined variables when establishing the model: diet and antibiotic use. Because diet is a key determinant of gut microbial ecology, we modeled a major feature of the western lifestyle by feeding mice a fiber-deficient (FD) diet (28, 29) . Most adults in the United States fail to meet recommended daily fiber intake (30), and this chronic deficiency alters both the microbiota and the metabolic environment of the gastrointestinal tract. Therefore, we rationalized that the FD diet allowed us to mimic the nutritional context in which GBS is likely to occur in the GI tract of adults in the United States and other parts of the industrialized world. In addition, antibiotic usage is not a risk factor for GBS carriage (24), suggesting that GBS can invade human microbiomes in the absence of large-scale ecological disturbances. Therefore, we did not pre-treat mice with antibiotics prior to inoculation with GBS.

**Fig 1:**
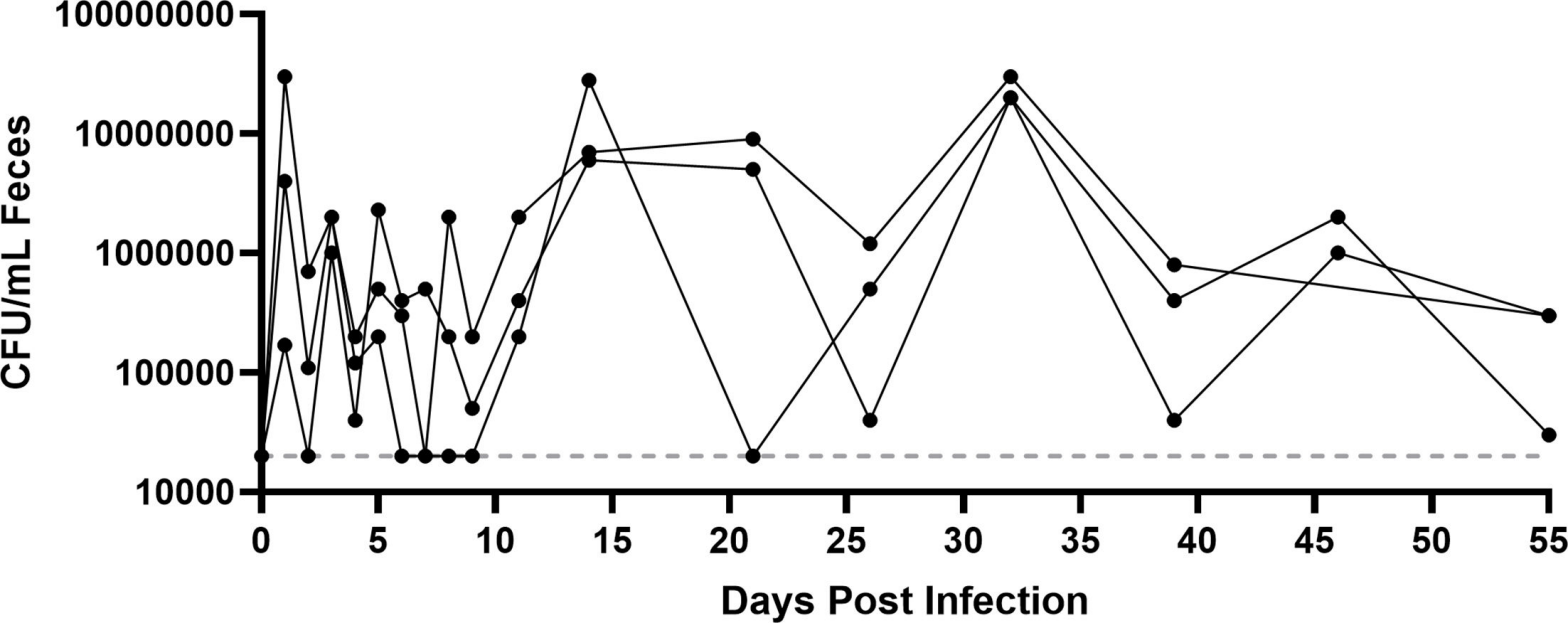
Long-term gastrointestinal colonization of mice with GBS. Female C57BL/6 mice (*n* = 3) were maintained on a fiber-deficient diet for 1 week prior to inoculation with 1e7 CFU GBS strain COH1 via droplet feeding (see Methods). Feces were collected from the mice at the indicated time points, and GBS was enumerated on Granada agar. Lines represent data collected from individual mice over time. The horizontal dotted line represents the limit of detection of the GBS plating assay (20,000 CFU/mL feces).

After inoculation of non-antibiotic-treated mice fed the FD diet with GBS strain COH1, we observed long-term colonization of the murine GI tract for up to 2 months with fluctuations in overall GBS abundance, as determined by fecal sampling (Fig. 1). Mice showed no behavioral signs of disease, such as decreased activity, hunched posture, ruffled fur, or changes to feeding and drinking habits, at any time point. Together, these observations are consistent with long-term asymptomatic carriage of GBS.

Because a common concern in murine GI models is that coprophagy could artificially maintain fecal burdens of microbes that poorly colonize, we housed mice individually on wire bottom cages to limit coprophagy and found no difference in GBS burdens compared to control mice that we co-housed under standard husbandry conditions (Fig. S1), confirming that GBS GI colonization in the mice represents a stable population rather than repeated re-inoculation with transiently colonizing GBS. We also tested whether host biological sex influenced colonization and found no difference in GBS burdens between male and female mice (Fig. S2), consistent with human survey data indicating similar GBS carriage between males and females (24). These results establish that our model recapitulates robust, asymptomatic GBS carriage in the adult GI tract, independent of coprophagy or host biological sex.

### Generalizability of the model to other GBS strains

GBS strains are commonly classified into 10 recognized serotypes (Ia, Ib, II–IX) (31, 32), with serotype distribution in asymptomatic screening and invasive disease varying geographically (33–35). In the US, the most common serotypes detected in human surveys are Ia, Ib, III, and V (7, 36, 37). COH1 is a serotype III clinical isolate commonly used in laboratory research (38–40) (Fig. 1). To test the generalizability of GBS GI colonization to other strains commonly used in GBS research (which also mirror serotypes commonly detected in the US), we colonized mice with GBS strains COH1, A909 (serotype Ia) (39, 41), and two serotype V strains (CJB111 (42, 43) and the hypervirulent CNCTC 10/84 (38, 39).

**Fig 2:**
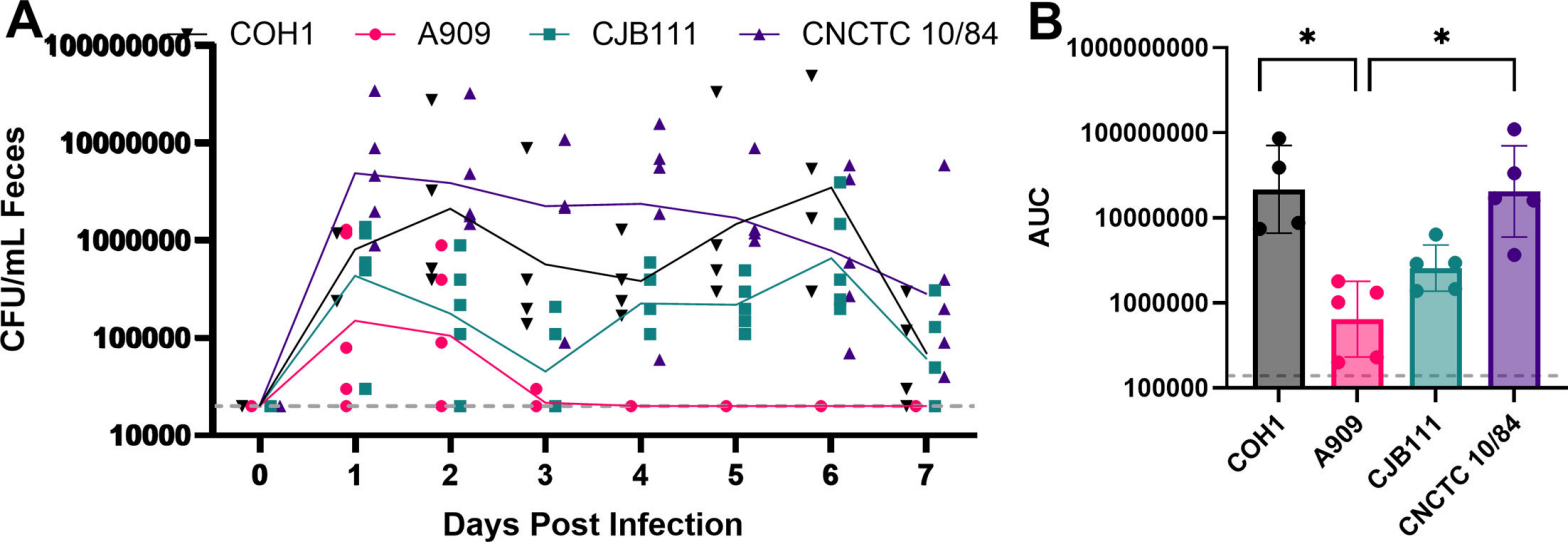
Gastrointestinal colonization with diverse GBS strains. Female C57BL/6 mice (*n* = 4 for COH1, *n* = 5 per group for all others) were maintained on a fiber-deficient (FD) diet for 1 week prior to inoculation with 1e7 CFU GBS strains COH1, A909, CJB111, or CNCTC 10/84 via droplet feeding (see Methods). (**A**) Feces were collected from the mice at the indicated time points, and GBS was enumerated on Granada agar. Points represent individual mice at each time point, and the line represents the geometric mean of GBS burden per strain per day. The horizontal dotted line represents the limit of detection of the GBS plating assay (20,000 CFU/mL feces). (**B**) Area under the curve (AUC) from 0 to 7 days post-infection. Points represent individual mice; bar represents geometric mean; and error bars represent geometric standard deviation. The horizontal dotted line represents the lower limit AUC (140,000). Statistical significance was determined by the Kruskal-Wallis test and Dunn’s multiple-comparison test (*= *P*-value < 0.05).

Like COH1, we observe that CJB111 and CNTC 10/84 can persistently colonize the GI tract over the length of the experiment, while A909 has low GBS levels that decrease below the limit of detection within 4 days of inoculation (Fig. 2A). Comparison of the average area under the curve (AUC) for fecal CFU counts further illustrates this difference and reinforces the differences in colonization between strains (Fig. 2B). Together, these findings support the idea that genetic variation between GBS strains contributes to GBS fitness in the adult GI tract, as has been observed in the neonatal GI tract (44).

### Genome-wide analysis of GBS factors required for survival in the murine gastrointestinal tract

We next sought to identify which genetic factors GBS requires to colonize and survive in the murine GI tract. We utilized an existing GBS Tn mutant library (45–48) in the CJB111 (Serotype V) background (42, 43) to colonize the murine GI tract. Three groups of mice (six per group) were colonized with 1e7 CFU of the GBS Tn library in biological triplicate. Mice were monitored daily for signs of illness, and fecal samples were collected daily (Fig. S3A and B). At 7 days post-colonization, mice were euthanized, and cecal content was collected (Fig. S3C). The input and cecum recovered libraries were processed as described in the Materials and Methods.

**Fig 3:**
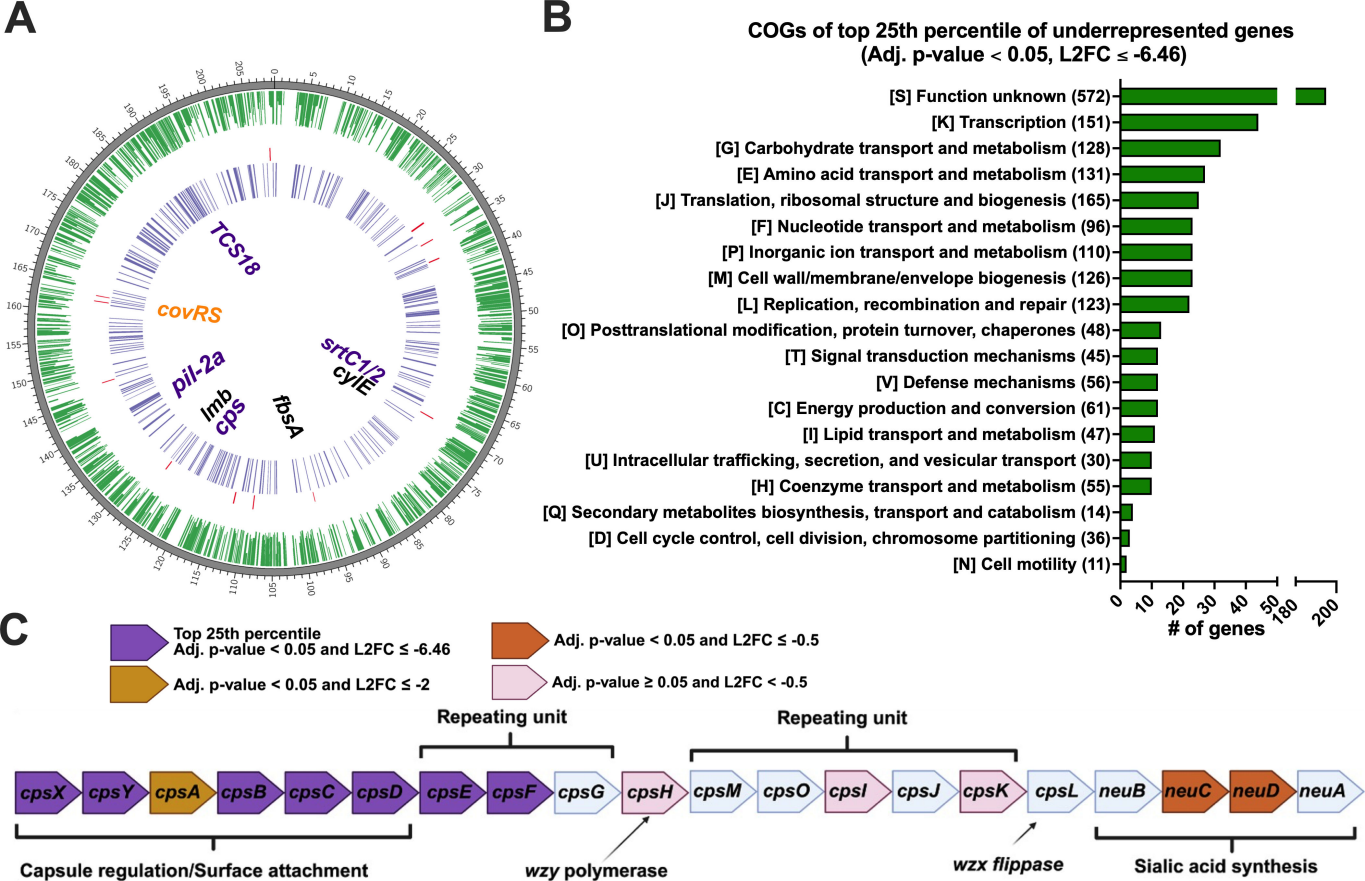
*In vivo* transposon-sequencing (Tn-Seq) of GBS CJB111 highlights genes possibly involved in GI colonization. (**A**) CIRCOS plot showing from outer ringer to inner ring; dark gray, genome length in Kb; green, statistically significant underrepresented genes (adj. *P*-value < 0.05 with Log2Fold change (L2FC) ≤ −2) ; red, statistically significant overrepresented genes (adj. *P*-value < 0.05 with L2FC ≥ 2); purple, genes in the top 25th percentile (adj. *P*-value < 0.05 with L2FC ≤ −6.46). Select genes are indicated in the center: top 25% of significantly underrepresented genes are labeled purple; other significantly underrepresented genes are labeled in black; and nonsignificant genes are labeled in orange. (**B**) COG distribution of the top 25% of significantly underrepresented genes. (**C**) GBS type V capsule biosynthesis gene operon.

To identify transposon insertion sites, sequenced reads were mapped to the GBS CJB111 genome (GenBank accession CP063198), which identified 1,066 genes as significantly underrepresented (adj. *P*-value < 0.05, log2FC ≤ −2) and 12 genes as significantly overrepresented (adj. *P*-value < 0.05, log2FC ≥ 2) in the cecum compared to the input library (Fig. 3A; Table S1). Due to the large number of significantly underrepresented genes from this initial analysis, we restricted further analysis to genes in the top 25th percentile of significantly underrepresented genes (L2FC ≤ −6.46). These genes were assigned clusters of orthologous groups of proteins (COGs). COGs, including carbohydrate transport and metabolism, amino acid transport and metabolism, nucleotide transport and metabolism, inorganic ion transport and metabolism, and cell wall/membrane/envelope biogenesis, had the greatest number of genes in the top 25th percentile of significantly underrepresented genes (Fig. 3B). We identified multiple genes known to contribute to GBS infection and adherence as significantly underrepresented, such as genes that are involved in β-hemolysin/hemolytic pigment, two-component regulatory systems, pilus genes, as well as metal carbohydrate transport (Table 1; Table S1).

**TABLE 1:** Underrepresented mutants of interest identified by *in vivo* gastrointestinal TN-seq

Open reading frame	Gene name	Description	Log2FC
ID870_03480	*cpsY*	LysR family transcriptional regulator	−8.15
ID870_03510	*cpsF*	UDP-N-acetylglucosamine--LPS N-acetylglucosamine transferase	−7.91
ID870_03490	*cpsB*	Tyrosine-protein phosphatase	−7.73
ID870_03495	*capA/cpsC*	Capsular polysaccharide biosynthesis protein	−7.38
ID870_03500	*cpsD*	Tyrosine-protein kinase	−7.33
ID870_00125	*phoU*	Phosphate signaling complex protein	−7.31
ID870_00130	TCS18_R	Response regulator transcription factor	−7.84
ID870_00135	TCS18_S	Two-component sensor histidine kinase	−7.33
ID870_06015	*srtC2*	Class C sortase	−7.46
ID870_06020	*srtC1*	Class C sortase	−7.26
ID870_02610	*srtC4*	PI-2a pilus assembly sortase	−6.75
ID870_02595	*pilA*	PI-2a pilus adhesin	−6.74
ID870_02600	*pilB*	PI-2a pilus major subunit	−6.69
ID870_02615	*pilC*	PI-2a pilus subunit	−6.24
ID870_05925	*cylE*	cylE protein	−2.76
ID870_03205	*lmb*	Metal ABC transporter substrate-binding lipoprotein/laminin-binding adhesin	−5.12
ID870_04105	*fbsA*	Fibrinogen-binding adhesin	−6.14

Among the most significantly underrepresented genes were genes of the capsular polysaccharide synthesis locus. The type V capsule biosynthesis pathway of CJB111 is displayed in Fig. 3C (49). The genes, *cpsXYABCD*, are putative capsule regulation and surface attachment genes, with five out of the six being in the top 25th percentile of genes in the dataset (Fig. 3C). Additional genes in the top 25th percentile were *cpsEF*, which were putatively involved in the repeating unit biosynthesis (Fig. 3C). Taken as a whole, this suggests the importance of capsule in GI survival, where the loss of any step in the biosynthesis pathway leads to decreased GBS survival.

### Loss of capsule leads to decreased GBS survival in the adult mouse GI tract

**Fig 4:**
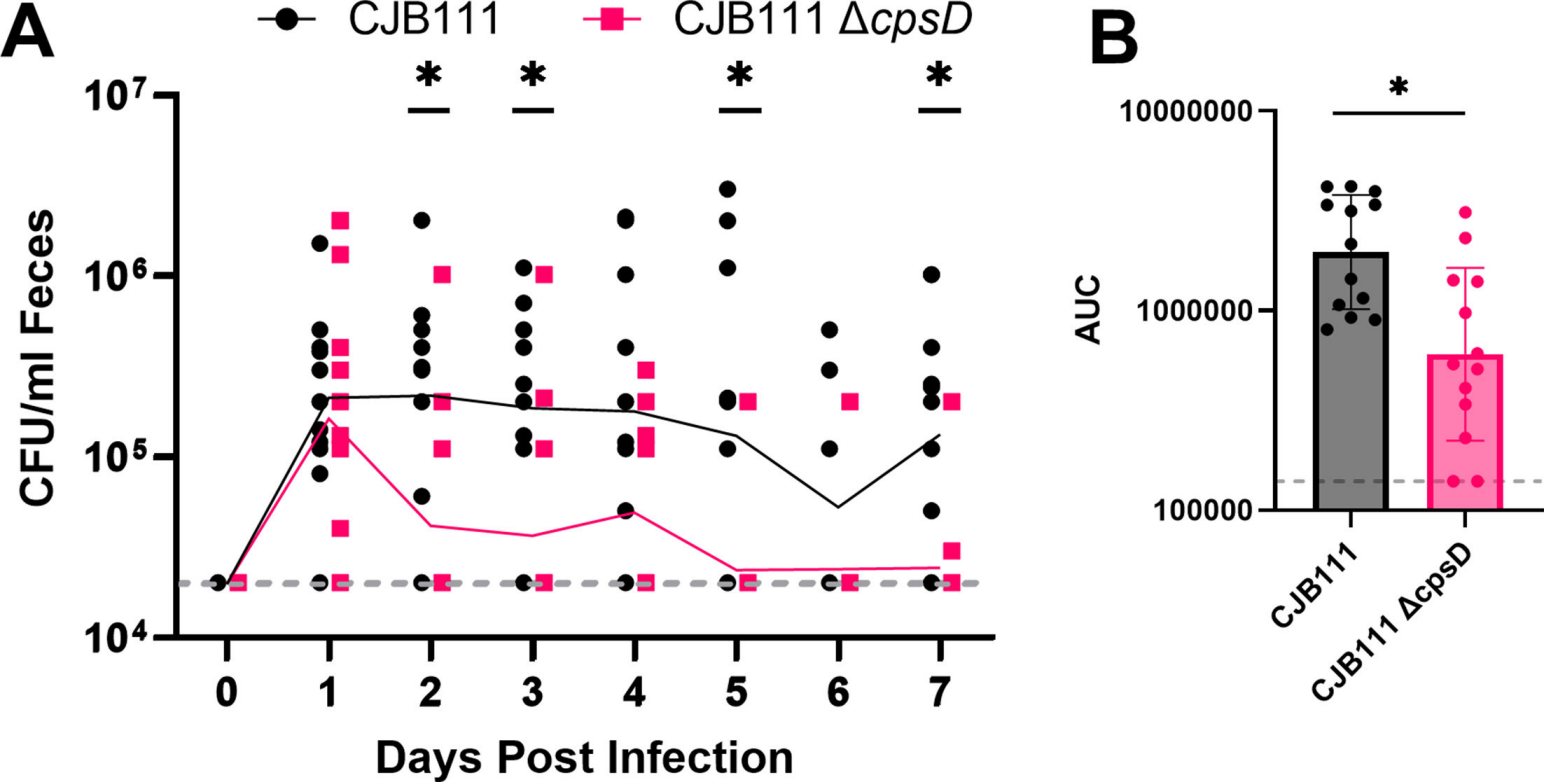
Capsular polysaccharide is important for GBS colonization of the adult mouse GI tract. Male and female C57BL/6 mice (*n* = 13 per group) were maintained on a fiber-deficient (FD) diet for 1 week prior to inoculation with 1e7 CFU GBS strains CJB111 WT or CJB111 ΔcpsD via droplet feeding (see Methods). (**A**) Feces were collected from the mice at the indicated time points, and GBS was enumerated on Granada agar. Points represent individual mice at each time point, and the line represents the geometric mean of the GBS burden. The horizontal dotted line represents the limit of detection of the GBS plating assay (20,000 CFU/mL feces). (**B**) Area under the curve (AUC) from 0 to 7 days post-infection. Points represent individual mice; bars represent geometric mean; and error bars represent geometric standard deviation. The horizontal dotted line represents the lower limit AUC (140,000). Mann-Whitney test (*= *P*-value < 0.05) showed statistically discernible differences between AUC of the strains.

The Tn-seq experiment identified mutants in the capsule biosynthesis locus as being significantly underrepresented in the strain library present in cecal contents. To test the importance of capsule in GBS GI colonization, the colonization experiment was repeated using a CJB111 ∆*cpsD* mutant, which has reduced abundance of capsule compared to WT (45). Daily fecal sampling shows the capsule mutant is less fit in the GI tract, with the majority of ∆*cpsD*-colonized mice having GBS burdens below the limit of detection by the end of the sampling period (Fig. 4A). Comparison of the average area under the curve (AUC) for fecal CFU counts from mice colonized with CJB111 or CJB111 ∆*cpsD* further reinforces the role that capsule plays in colonization of the murine adult GI tract (Fig. 4B). Taken together, these observations support that the capsule is important in maintaining colonization of the adult GI tract.

## DISCUSSION

Group B *Streptococcus* is a serious health concern for humans across their lifespan. While current antibiotic-based interventions and treatments have been effective, the rise in antibiotic resistance and increasing awareness of the negative impacts of antibiotic-mediated microbiome disruption emphasize the need for non-antibiotic approaches for mitigating GBS disease. In addition, the majority of invasive GBS diseases have been in non-pregnant adults in recent years. Unlike neonates and pregnant people, there does not exist a standard prophylaxis approach for non-pregnant individuals who predominantly acquire GBS by community spread. More research is required to understand the community reservoir of GBS, the adult GI tract.

Here, we present a robust model of GBS adult GI colonization that paves the way for a better understanding of this pathogen in this important but understudied niche and established that capsular polysaccharide is an important GBS-encoded molecular factor required for colonization. This contributes to a growing body of literature that highlights the importance of capsule for GBS to colonize and cause disease in a variety of host-associated niches (44, 50, 51). We observed differences in the fitness of different GBS strains, with A909 (Ia serotype) not colonizing as well compared to COH1 (III), CJB111 (V), or CNTC 10/84 (V) (Fig. 2). Similar strain differences are also seen in a murine model of vaginal colonization, with A909 having lower colonization fitness compared to CJB111 and COH1 (52). CJB111 and COH1 have different capsular polysaccharide (CPS) structures, making this major surface-exposed macromolecule a likely contributor to differences in GI colonization. However, non-CPS genetic variability between the strains might affect these differences in colonization dynamics, highlighting an important area of future investigation.

The 10 antigenically distinct GBS CPS types are made up of varying linkages of glucose and galactose with a terminal side chain sialic acid (53). While the capsule regulatory genes and the sialic acid synthesis genes are well conserved across the CPS types (53), differences in glycotransferases and resulting sugar structure could contribute to differences in strain ability to colonize in the GI and other body sites. Previous work in the neonatal murine gut showed that wild-type A909 was able to outcompete an isogenic mutant of A909 engineered to CPS III (44). This could suggest differences in the role that GBS capsular polysaccharide structure plays in the immature neonatal gut versus the adult gut.

Further supporting the importance of capsule in GI colonization, we showed via *in vivo* Tn-seq that multiple GBS CPS biosynthesis genes were in the top 25% percentile of underrepresented genes (Fig. 3). We then showed that a serotype V capsule mutant is unable to colonize the GI tract *in vivo* to the same extent as the WT (Fig. 4). More work will need to be done to further interrogate the role of CPS type on gastrointestinal colonization (e.g., adherence to mucus or epithelial cells).

Other genes significantly underrepresented in the output library include the sortases *srtC1* and *srtC2*, known adherence factors, such as PI-2a (54) and *lmb (*55, 56), that promote colonization and adherence to epithelial cells and extracellular matrix component. Notably, the CovR/S transcriptional regulation system, which has previously been identified as important in colonization and virulence (57, 58), was not significantly underrepresented. Instead, the two-component system TCS18 was in the top 25% of underrepresented COGs. TCS18 (i.e., PhoBR) seems to impact GBS biofilm formation and hemolysin production by binding to the promoter regions of *hylA* and *ciaR (*59). Previous CJB111 Tn-seq experiments done in other GBS body sites, such as the female reproductive tract (48), the bloodstream (46), and diabetic wound ulcers (45) also identified similar virulence factors and two-component systems as significantly underrepresented. Taken together, this suggests that the same genetic factors that enable colonization and virulence in other body sites could also be involved in establishing asymptomatic colonization in the GI tract.

A limitation of this study is that we focused solely on the large intestine (cecal contents and feces) and did not investigate GBS in other parts of the GI tract. This was done due to the large intestine having the largest amount of biomass in the GI tract. However, it is well known that the ecology of the microbiome varies widely down the length of the GI tract. *Streptococcal* species are hallmarks of the oral cavity and the small intestine (60, 61). While GBS has been isolated in the oral cavity (62), it is not the majority member. This could suggest that GBS is outcompeted by other *Streptococcal* species in these niches, making the GBS the preferred GI niche in the large intestine. Future work will leverage this model to establish GBS biogeography throughout the longitude of the GI tract and identify the microbial and host factors important for maintaining colonization in these diverse locations.

This work also lays the groundwork for further study into how diet impacts GBS in the GI. The GI tract is a mosaic of fluctuating nutrients, with host diet dictating availability and, by extension, impacting bacterial fitness. GBS, like other *Streptococcus* species, has a reduced genome of approximately 2 Mb. As a result, GBS is an auxotroph for many essential compounds and depends predominantly on scavenging from the environment and other bacterial community members. In addition to previously identified virulence factors and two-component regulatory systems, a variety of carbohydrate and amino acid transport/metabolism genes were underrepresented (Table S1). Notably, CJB111’s known glucosidases were not significantly underrepresented, which aligns with the absence of fiber in the diet. It is possible that these genes would be more crucial in different dietary conditions. In contrast, sugar uptake loci, such as *celAB* and *manN*, were underrepresented in our data set, suggesting that metabolism of simple sugars may be more critical for GBS persistence in the GI of mice on a fiber-deficient diet. *ManN* was also underrepresented in the vagina (48) and diabetic wound ulcer (45) Tn-seqs, suggesting a shared importance for GBS colonization and pathogenesis across body sites.

The amino acid metabolism genes *ilvE* (involved in branched-chain amino acid metabolism) and *glyA* (involved in serine metabolism) were also underrepresented in the GI. Differences in carbohydrate and amino acid genes underrepresented in the GI tract compared to the vagina (48), bloodstream (46), and diabetic wound ulcer (45) suggest the possibility of niche-specific metabolic activity. We anticipate that this model will enable focused research into how host diet could be leveraged as a non-invasive, non-antibiotic-based intervention to decrease GBS GI colonization rates and mitigate subsequent disease.

We anticipate that this model will also enable future study into how GBS transmits from the GI reservoir to other body sites, such as the urogenital tract, and to other at-risk individuals, such as neonates and immunocompromised adults. GBS adult GI colonization predisposes human urogenital tract colonization (27), and this model can be leveraged to explore the bacterial and host factors that drive this transmission. Additionally, longitudinal rectovaginal screening of pregnant people suggests that the GBS status can be transient (63), suggesting transient factors, such as host behavior and diet, could impact GBS burdens and risk of transmission. This is echoed in our own findings where we see day-to-day fluctuations in GBS burdens. Future work could aim to address the host and bacterial factors that drive this variability.

The adult GI tract is an understudied but vital reservoir for GBS. A better understanding of GBS physiology in the GI tract is crucial in the development of targeted interventions. We have established a robust adult mouse model that recapitulates key human host factors, such as being sex-independent and not requiring antibiotic pre-treatment. This model opens the door to a mechanistic dissection of how GBS persists in the gut and how this impacts carriage at other body sites, transmission to neonates, and subsequent neonatal and adult disease.

## MATERIALS AND METHODS

### Bacterial strains and culture conditions

GBS strains COH1 (serotype III), A909 (serotype Ia) (39, 41), CJB111 (serotype V) (42, 43), CNCTC 10/84 (serotype V) (38, 39), and CJB111 ∆*cpsD (*45) were maintained at −80°C as 25% glycerol stocks. Strains were routinely cultured on tryptic soy broth agar (TSB, Neogen) and incubated aerobically at 37°C. After overnight growth, a single colony was picked into 5 mL of TSB and grown aerobically at 37°C for 16–24 h. Liquid cultures were used as inocula for mouse experiments, as described below.

### Murine model of GBS GI colonization

All animal studies were carried out in strict accordance with the University of Wisconsin—Madison Institutional Animal Care and Use Committee (IACUC) guidelines (Protocol #M006305). Six-to-eight-week-old C57BL/6 mice were purchased from Taconic and used as in-house breeders. In-house bred mice between 6 and 15 weeks of age were used in experiments. Mice were fed a fiber-deficient (FD) diet (Inotiv TD.150689) starting 1 week before GBS inoculation.

Mice were inoculated via the oral droplet feeding method. This method was developed based on an existing murine model of *Klebsiella pneumoniae* gastrointestinal colonization (64). In brief, on the day of inoculation, food and water were withheld from mice 4 h before oral droplet feeding. To prepare the inoculum, 5 mL overnight GBS liquid culture was centrifuged at 4,000 × *g* for 15 min. The resulting bacterial pellet was resuspended and washed once with phosphate-buffered saline (PBS) and resuspended in equal volume of sterile 2% sucrose. Using this inoculum, mice were fed ~1e8 CFU/mL total of GBS split between two 50-μL doses an hour apart via a micropipette tip.

Feces were collected from mice directly into a microcentrifuge tube and kept on ice until plating, upon which they were stored at −80°C. To quantify GBS burdens, 1 µL of fecal sample was resuspended in 200 µL PBS in sterile polystyrene 96-well tissue culture plates (28, 29). Next, 10-fold serial dilutions were prepared, and 10 µL of each dilution was spread on Granada agar (65) (a differential and selective medium for GBS) with two technical replicates. Granada agar plates were incubated anaerobically for 24 h. Colonies were quantified, and the technical replicates were averaged to determine the GBS burden (limit of detection = 2e4 CFU/mL).

### Murine GI colonization Tn-seq

Triplicate 1 mL frozen aliquots of the pooled CJB111 pKrmit transposon library (47) were thawed and resuspended in 4 mL TSB with kanamycin at 300 µg/mL and grown overnight at 37°C. Cultures were washed in PBS, and then normalized to 1e8 CFU/mL in 2% sucrose in sterile PBS as described above. Mice were then fed the library via oral droplet feeding as described above. Next, 100 μL of the input libraries was spread on CHROMagar Strep B with 300 µg/mL kanamycin in triplicate and incubated overnight at 37°C. Burdens of GBS were monitored via fecal sampling as described above with the exception that fecal samples were plated on CHROMagar Strep B with 300 µg/mL kanamycin. Seven days post-infection, mice were euthanized, and cecal content was collected. Cecal content was resuspended in 1 mL sterile PBS and spread on CHROMagar Strep B with 300 µg/mL kanamycin in triplicate and incubated overnight at 37°C to collect recovered transposon mutants. Bacterial growth from spread plates was collected using a sterile tissue scraper and the six mice per library pooled together. The pooled recovered transposon mutants were centrifuged at 4,000 × *g* for 15 min. Supernatant was then removed, and bacterial pellets were then frozen and stored at −80°C before genomic DNA was extracted using the phenol-chloroform method (66).

### Transposon library sequencing

Libraries were prepared and sequenced at the University of Minnesota Genomics Center (UMGC) according to https://www.protocols.io/view/transposon-insertion-sequencing-tn-seq-library-pre-rm7vzn6d5vx1/v1. Briefly, genomic DNA was enzymatically fragmented; adapters were added using the NEB Ultra II FS Kit (New England Biolabs); and ~50 ng of fragmented adapted gDNA was used as a template for enrichment by PCR (16 cycles) for the transposon insertions using mariner-specific (TCGTCGGCAGCGTCAGATGTGTATAAGAGACAGCCGGGGACTTATCATCCAACC) and Illumina P7 primers. The enriched PCR products were diluted to 1 ng/μL, and 10 μL was used as a template for indexing PCR (nine cycles) using Nextera_R1 (iP5) and Nextera_R2 (iP7) primers. Sequencing was performed using 150 base paired-end format on the Element Aviti System to generate ~40 million reads per library.

### Tn-seq bioinformatic analyses

Bioinformatic analyses were performed as previously described with minor modifications (45, 46). TRANSIT2 (v 1.1.7) (67) was used to trim reads and align them to the CJB111 genome (CP063198) for analysis of transposon insertion sites. The Transit PreProcessor processed reads using default parameters with the Sassetti protocol, primer sequence ACTTATCAGCCAACCTGTTA, and mapped them to the genome using Burrows-Wheeler Alignment (BWA) (68). Insertion sites were normalized using the beta-geometric correction (BGC) in TRANSIT2 and analyzed using the site-restricted resampling, which was performed using default parameters, with the addition of ignoring TA sites within 5% of the 5′ and 3′ end of the gene, to compare the insertion counts recovered from the cecum vs the input library. All sequencing reads have been deposited into National Center for Biotechnology Information SRA under BioProject accession: PRJNA1328725.

### Statistical analysis

All statistical analysis, except those done for the Tn-seq (see above), were performed using GraphPad Prism 10. Specific statistical analyses are noted in the relevant figure legends.

## SUPPLEMENTAL MATERIAL

Fig. S1 iai.00527-25-s0001.tif GBS GI carriage is independent of coprophagy.

Fig. S2 iai.00527-25-s0002.tif Biological sex does not impact the GBS GI carriage.

Fig. S3 iai.00527-25-s0003.tif Fecal and cecal burdens of the GBS CJB111 pKrmit transposon strain.

Supplemental material iai.00527-25-s0004.docx Supplemental figure legends.

Table S1 iai.00527-25-s0005.xlsx *In vivo* GBS CJB111 Tn-Seq screen.

ASM does not own the copyrights to Supplemental Material that may be linked to, or accessed through, an article. The authors have granted ASM a non-exclusive, world-wide license to publish the Supplemental Material files. Please contact the corresponding author directly for reuse.
