# Supplementary figures and images for: A murine model of adult gastrointestinal colonization by Group B Streptococcus
Source: Infect Immun. 2026 Jan 30;94(3):e00527-25. doi: 10.1128/iai.00527-25 (PMC12974117; doi:10.1128/iai.00527-25)

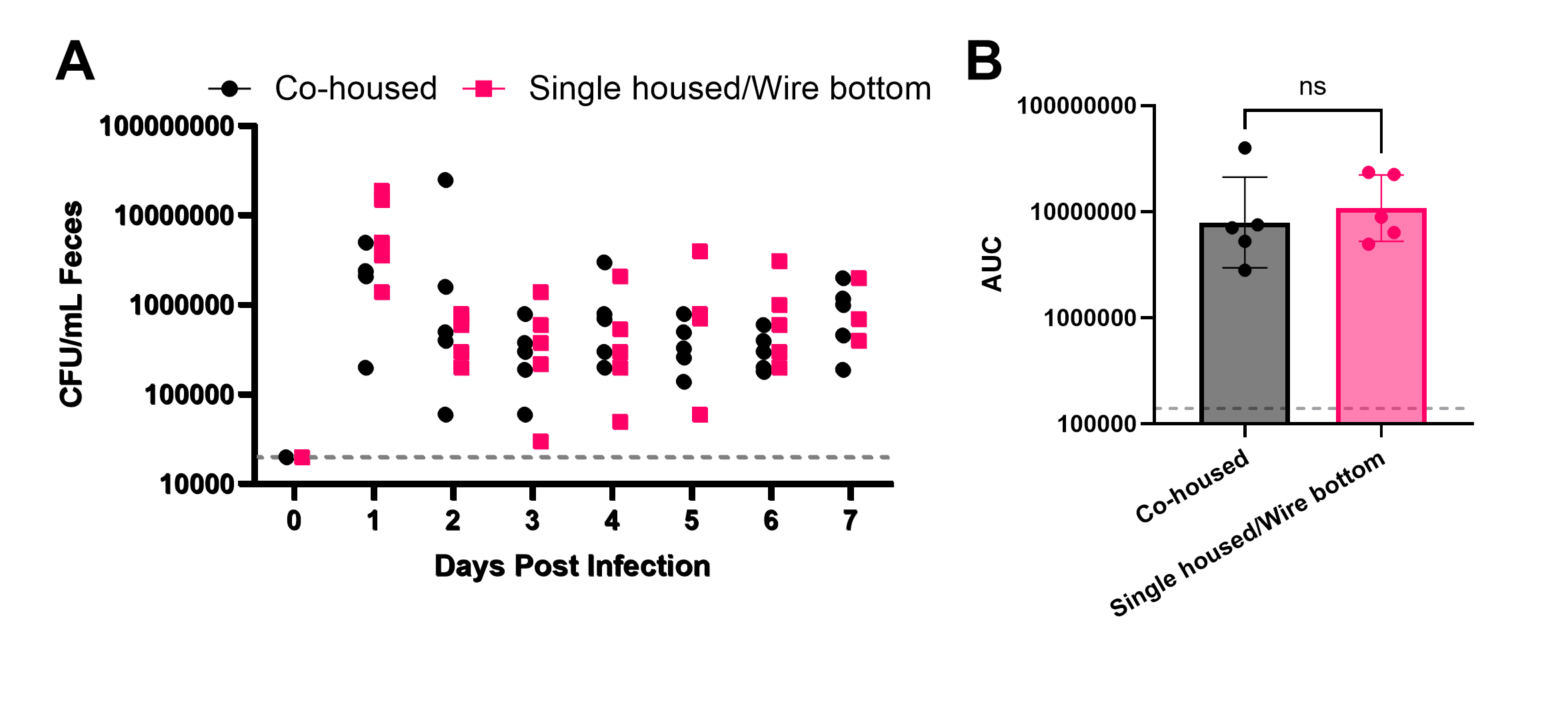

Supplement: Fig. S1 — GBS GI carriage is independent of coprophagy. [file iai.00527-25-s0001.tif]

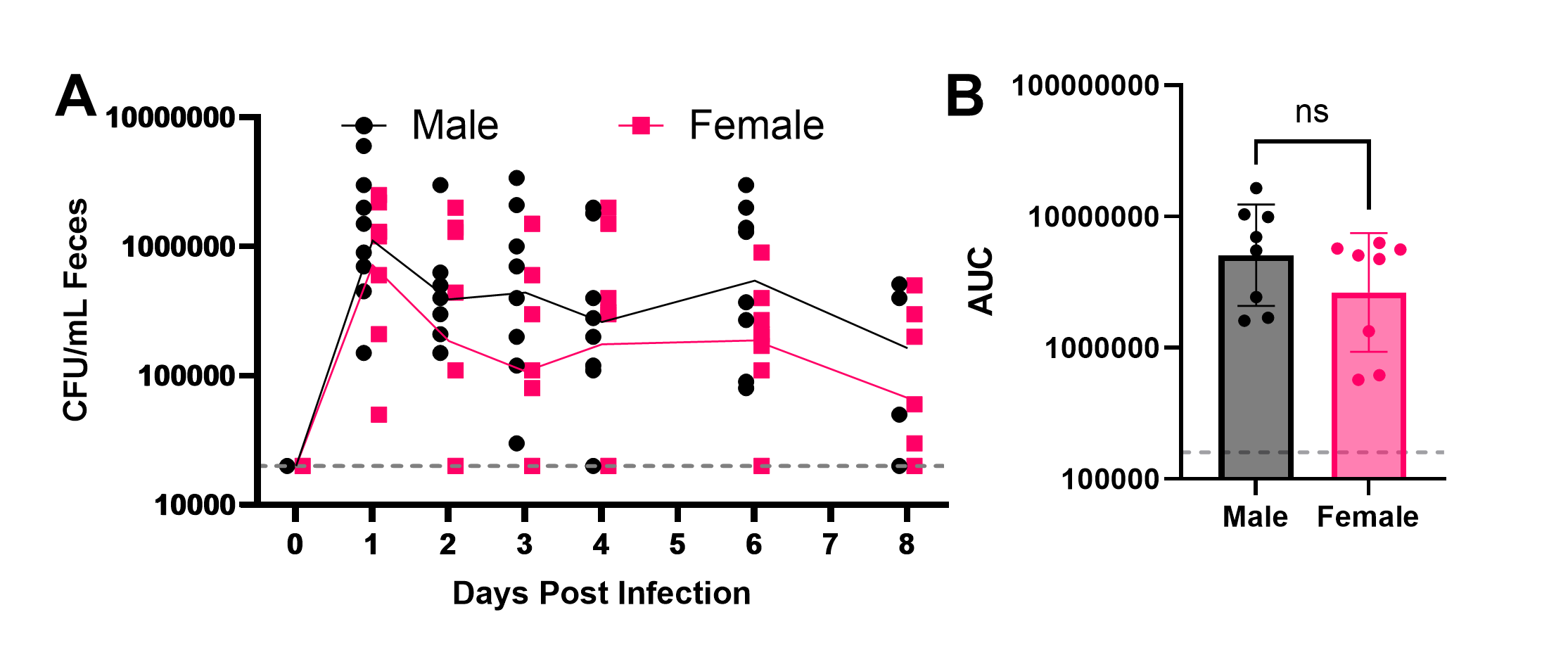

Supplement: Fig. S2 — Biological sex does not impact the GBS GI carriage. [file iai.00527-25-s0002.tif]

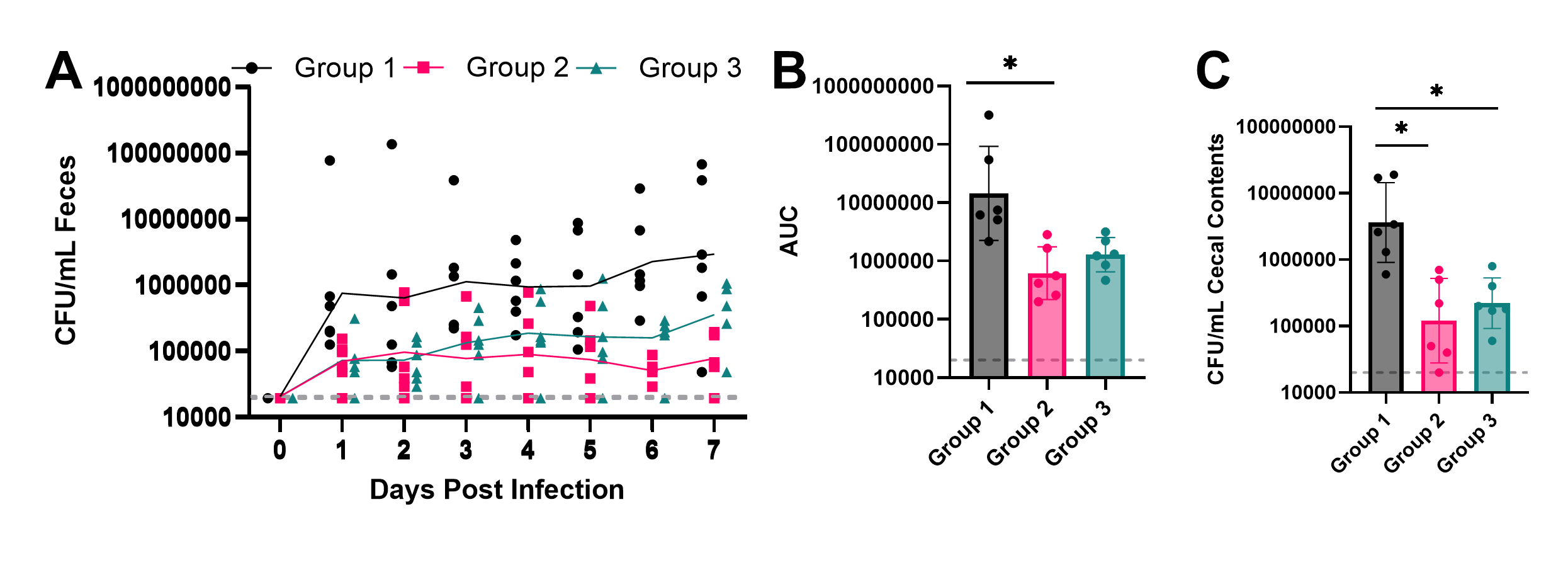

Supplement: Fig. S3 — Fecal and cecal burdens of the GBS CJB111 pKrmit transposon strain. [file iai.00527-25-s0003.tif]
