# Supplementary material for: A murine model of adult gastrointestinal colonization by Group B Streptococcus
Source: Infect Immun. 2026 Jan 30;94(3):e00527-25. doi: 10.1128/iai.00527-25 (PMC12974117; doi:10.1128/iai.00527-25)
Supplement: Supplemental material — Supplemental figure legends. [file iai.00527-25-s0004.docx]

**Supplementary Figure Legends**

Supplementary Figure 1: GBS GI carriage is independent of coprophagy. Male C57BL/6 mice (n=5 per group) were maintained on a fiber deficient (FD) diet for 1 week prior to inoculation with 1e7 CFU GBS strain COH1 via droplet feeding (see Methods). **(A)** Feces were collected from the mice at the indicated time points and GBS was enumerated on Granada agar. Points represent individual mice at each time point and line represents geometric mean of GBS burden. The horizontal dotted line represents the limit of detection of the GBS plating assay (20,000 CFU/mL feces). Mann-Whitney test (*= p-value<0.05) showed no statistically discernible differences between fecal CFU/mL of the two strains. **(B)** Area under the curve (AUC) from 0 to 7 days post infection. Points represent individual mice, bars represent geometric mean, and error bars represent geometric standard deviation. The horizontal dotted line represents the lower limit AUC (140,000). Mann-Whitney test (*= p-value<0.05) showed no statistically discernible differences between AUC of the strains.

Supplementary Figure 2: Biological sex does not impact GBS GI carriage. Male and female mice (n=8 per group) were maintained on a fiber deficient (FD) diet for 1 week prior to inoculation with 1e7 CFU GBS strain COH1 via droplet feeding (see Methods). **(A)** Feces were collected from the mice at the indicated time points and GBS was enumerated on Granada agar. Points represent individual mice at each time point and line represents geometric mean of GBS burden. The horizontal dotted line represents the limit of detection of the GBS plating assay (20,000 CFU/mL feces). Mann-Whitney test (*= p-value<0.05) showed no statistically discernible differences between fecal CFU/mL of the two biological sexes. **(B)** Area under the curve (AUC) from 0 to 8 days post infection. Points represent individual mice, bars represent geometric mean, and error bars represent geometric standard deviation. The horizontal dotted line represents the lower limit AUC (160,000). Mann-Whitney test (*= p-value<0.05) showed no statistically discernible differences between AUC of the strains.

Supplementary Figure 3**:** Fecal and cecal burdens of GBS CJB111 pKrmit transposon strain. Male and female mice (n=6 per group) were maintained on a fiber deficient (FD) diet for 1 week prior to inoculation with 1e7 CFU GBS strain COH1 via droplet feeding (see Methods). **(A)** Feces were collected from the mice at the indicated time points and GBS was enumerated on CHROMagar Strep B with 300 µg/mL kanamycin. Points represent individual mice at each time point and line represents geometric mean of GBS burden. The horizontal dotted line represents the limit of detection of the GBS plating assay (20,000 CFU/mL feces). **(B)** Area under the curve (AUC) from 0 to 7 days post infection. Points represent individual mice, bar represents geometric mean, and error bars represent geometric standard deviation. The horizontal dotted line represents the lower limit AUC (140,000). Kruskal-Wallis test and Dunn’s multiple comparison test (*= p-value<0.05) showed statistically discernible differences between AUC of the three cages. **(C)** Cecal content was collected at 7 days post infection and GBS was enumerated on CHROMagar Strep B with 300 µg/mL kanamycin. Points represent individual mice, bars represent geometric mean, and error bars represent geometric standard deviation. The horizontal dotted line represents the limit of detection of the GBS plating assay (20,000 CFU/mL feces). Kruskal-Wallis test and Dunn’s multiple comparison test (*= p-value<0.05) showed statistically discernible differences between AUC of the three cages.
